# Effectiveness of a Group-Based Psychological Safety Intervention to Prevent Workplace Bullying and Sustain Work Engagement: A Cluster Randomized Controlled Trial

**DOI:** 10.3390/bs15101302

**Published:** 2025-09-24

**Authors:** Momoko Kobayashi

**Affiliations:** Center for Well-Being Support, Saga University, 1 Honjo-machi, Saga 840-8502, Japan; kobamo@cc.saga-u.ac.jp

**Keywords:** psychological safety, workplace bullying, work engagement, stress-coping strategies, interpersonal communication, cluster randomized controlled trial, employee well-being, organizational intervention

## Abstract

Building psychological safety is vital for preventing workplace bullying and for sustaining employee well-being, organizational performance, and work engagement. This study developed and evaluated a short-term intervention promoting psychological safety and communication skills. The program provided strategies for addressing during high-stress situations and fostered a shared understanding of honest communication. A cluster randomized controlled trial was conducted in a pharmaceutical company, with departments assigned to intervention or control groups. The effects were examined using a generalized linear mixed-effect model. In the intervention group, psychological safety with supervisors and teams significantly increased, preceding a significant rise in consultation tendencies, whereas bullying and supervisor-related intimidation significantly decreased. Work engagement declined in the control group but remained stable in the intervention group. These findings demonstrate that the program enhanced psychological safety, enabling employees to express opinions without irrational fear, which in turn increased consultation, improved communication, reduced bullying, and sustained work engagement. The group-based format further emphasized mutual understanding and skill-sharing, providing broad organizational benefits. This study positions psychological safety as a proximal mechanism that facilitates consultation behaviors and demonstrates the feasibility of integrating brief, evidence-based training into routine organizational development.

## 1. Introduction

Workplace bullying and employee well-being have increasingly become central topics in occupational health psychology, with growing evidence highlighting their profound impact on individual health and organizational functioning. Workplace bullying—commonly referred to as power harassment in Japan—has been the leading cause of individual labor dispute consultations for the past decade. It also ranked as the most common cause of mental disorder-related workers’ compensation claims in 2021, underscoring its status as a pressing social issue. Workplace bullying not only harms physical and mental health (Leymann & Gustafsson, 1996; Mikkelsen & Einarsen, 2002; Kivimäki et al., 2003; Niedhammer et al., 2006) but also causes indirect damage such as a deteriorating work environment, reduced productivity, and increased turnover intentions (Nielsen & Einarsen, 2012). In contrast, higher work engagement is associated with better physical and mental health, stronger commitment and performance, and lower turnover intentions (Hakanen et al., 2008; Shimazu et al., 2015), benefiting both employees and organizations. Despite the urgent need for preventive measures and intervention research, few studies in Japan have demonstrated effective workplace bullying interventions. Most occupational stress research has traditionally focused on issues such as overwork and extended working hours, with limited practical or workplace-based intervention studies. Similarly, most work engagement studies remain theoretical (Bakker & Leiter, 2010), and few have validated effective strategies for improving engagement in practice.

Workplace bullying and work engagement reflect contrasting workplace experiences: bullying undermines health and motivation, whereas engagement is associated with energy and fulfillment. Although typically examined individually, recent studies have emphasized the importance of simultaneously examining these phenomena. Studies in other and non-western countries indicate that exposure to bullying is associated with decreased engagement and other related outcomes (e.g., Javed et al., 2023; Park & Ono, 2016). However, empirical evidence in Japan remains scarce. Kobayashi et al. (2024) conducted one of the first evaluations of manager-focused intervention that targeted power harassment and found that participants who reported reduced harassment also tended to exhibit increased engagement. Although promising, systematic reviews stress that the overall quality of intervention evidence remains limited and underscore the need for further rigorous, controlled trials (Ruotsalainen et al., 2015).

Parallel efforts that focus on engagement have also yielded mixed results in Japan. For instance, the benefits of job crafting or web-based programs were mainly observed among subgroups with initially low levels of engagement, indicating potential ceiling effects and the importance of contextual moderators (Sakuraya et al., 2020; Imamura et al., 2017). These findings highlight the role of organizational climate and mechanisms that operate across bullying and engagement. In particular, research on psychosocial safety climate and psychological safety illustrates that shared perceptions of support and openness foster engagement while discouraging bullying behaviors (Inoue et al., 2023; Dollard & Bakker, 2010).

Taken together, the existing evidence implies that (a) bullying and engagement are still rarely examined together, (b) robust intervention research in non-western contexts, including Japan, remains underdeveloped, and (c) organization-embedded approaches that focus on shared antecedents, such as psychological safety, are especially warranted. To address these research gaps, this study develops and evaluates the effectiveness of a short-term, group-based program designed to enhance psychological safety, prevent bullying, and promote work engagement among employees in Japan.

Preventing bullying is expected not only to mitigate its harmful effects but also to help maintain and enhance work engagement (Kobayashi et al., 2024). Based on previous research (Law et al., 2011), fostering psychological safety is a shared approach to achieving both outcomes. Psychological safety refers to a team’s shared belief that the workplace is safe for interpersonal risk-taking (A. Edmondson, 1999), where individuals can express opinions openly without fear of rejection, punishment, or conflict. Such environments promote mutual respect, openness, and constructive dialogue, enhancing learning behaviors, engagement, and organizational performance (A. Edmondson, 1999). Psychological safety is also crucial for long-term organizational sustainability. Although practical efforts to enhance psychological safety are underway in Japan, there remains a lack of evidence-based intervention studies, signaling the need for further research (Koutani et al., 2025).

The openness of managers and leaders, their approachability, and their ability to create opportunities for dialogue are regarded as key antecedents of psychological safety (A. C. Edmondson et al., 2004). Many practices aimed at enhancing psychological safety focus on managerial behavior. However, research suggests that among Japanese workers, the mere permission to express opinions does not necessarily translate into actual expression (Ochiai & Otsuka, 2022). Therefore, in addition to managerial openness and empathy, subordinates must also develop the skills and willingness to communicate their ideas and opinions. Psychological safety is associated with factors such as a proactive approach to problem solving, high stress tolerance, emotional stability, supportive interpersonal relationships, and interdependence in collaborative tasks (A. C. Edmondson & Lei, 2014). Based on this, it is suggested that coping strategies for proactive problem-solving, effective communication skills across various workplace contexts, and the cultivation of mutually supportive relationships through consultation and requests contribute to a high level of psychological safety. In turn, this promotes the prevention of workplace bullying and the enhancement of work engagement. Since psychological safety reflects a group-level belief, shared within organizations or teams, that one can speak openly and without fear (A. C. Edmondson & Lei, 2014), programs should be implemented at the group level rather than the individual level, fostering a shared understanding.

Therefore, this study developed an intervention program for general employees aimed at building psychological safety to prevent workplace bullying and enhance work engagement. I assessed its multifaceted effects among workers at a pharmaceutical company using a cluster randomized controlled trial.

### Theoretical Framework

This study integrates psychological safety theory, the job demands–resources model (JDR), and stress and coping theory into a unified framework to explain the interaction of team climate and individual resources with workplace outcomes. Psychological safety is a team-level climate resource that enables self-expression, learning, and help-seeking (A. Edmondson, 1999; A. C. Edmondson & Lei, 2014). Within the JDR framework, psychological safety and consultative behaviors are conceptualized as collective and interpersonal job resources that sustain engagement, whereas workplace bullying is viewed as a hindrance demand that drains energy and impairs well-being (Demerouti et al., 2001; Bakker & Demerouti, 2007; Law et al., 2011). Hindrance demands are job demands that obstruct personal growth or goal attainment and thus undermine motivation and well-being. Stress and coping theory further elucidate that individual-level processes, such as training in cognitive reframing, self-regulation, and assertive communication, equip employees with personal and interpersonal coping strategies that can be used within stressful contexts (Lazarus & Folkman, 1984). These enhanced resources are expected to strengthen team climate, boost psychological safety, mitigate conditions that foster bullying, and maintain engagement under strain. Taken together, this integrated framework justifies the study’s cluster-level design (teams/departments) and the selection of outcomes that focus on bullying, psychological safety, consultative behaviors, and engagement.

## 2. Materials and Methods

### 2.1. Participants and Study Design

The survey and intervention were conducted with the cooperation of a pharmaceutical company, referred to here as Plant A. Plant A is one of the company’s pharmaceutical manufacturing sites and includes five major divisions: manufacturing, production engineering, quality control, quality assurance, and administration. The manufacturing sector in Japan is known for its high incidence of mental health issues (Ministry of Health, Labour and Welfare, 2023). In pharmaceutical production, the demands for quality, cost reduction, and supply stability contribute to high work stress. Plant A was considered an appropriate target for the survey because high-stress environments are more prone to power harassment (Baillien et al., 2011), and limiting the study to a single site helped control for regional and organizational differences. No conflict of interest is declared. All employees at Plant A were surveyed at three time points: July 2023, November 2023, and February 2024. Given that the study examined intervention effects on indicators that are closely related to organizational climate, such as workplace bullying and psychological safety, combining intervention and control groups within the same department was deemed inappropriate. Therefore, the study adopted a cluster randomized controlled trial (cRCT) design, in which departments were randomly assigned to the intervention or control groups. Compared with individual-level randomization, the cRCT design decreased the risk of contamination between participants within the same organizational unit, thereby enhancing the internal validity of the intervention effects. Moreover, randomization at the cluster level was theoretically more appropriate than individual-level allocation because the foci of the intervention program were group-level communication and organizational climate. At the same time, the study recognizes the limitations of cRCTs, including decreased statistical power due to intra-cluster correlation and the possibility of baseline imbalances between clusters. The study addressed these limitations by adjusting for clustering effects in the statistical analysis. Nevertheless, these limitations should be considered when interpreting the findings. Employees in intervention departments received a face-to-face program in August 2023 and an e-learning follow-up in September 2023. Surveys were conducted using Cuenote Survey, a cloud-based web questionnaire platform. The questionnaire explained the study’s purpose, voluntary participation, anonymity, lack of individual consequences, support resources for any issues, and plans for publication. Only participants who selected “I agree” proceeded with the survey. The study received ethical approval from the Kyushu University Ethical Review Committee (202112).

The number of subjects collected at each survey time point was as follows: Time 1—494 (valid responses: 392), Time 2—488 (394), and Time 3—488 (395). In this study, 368 participants were included in the analysis (Table 1), excluding those who did not consent, transferred departments during the study, or submitted incomplete responses. Given that the definition of power harassment in Japan explicitly requires it to occur against a background of workplace superiority, the study presents variables that could indicate superiority (i.e., gender, age, position, and years of service). Furthermore, given that this study classified employees according to organizational unit and conducted a cluster randomized trial, it also presented a breakdown by department. To characterize the pharmaceutical company under study, it describes the classification of job types.

**Figure 1:**
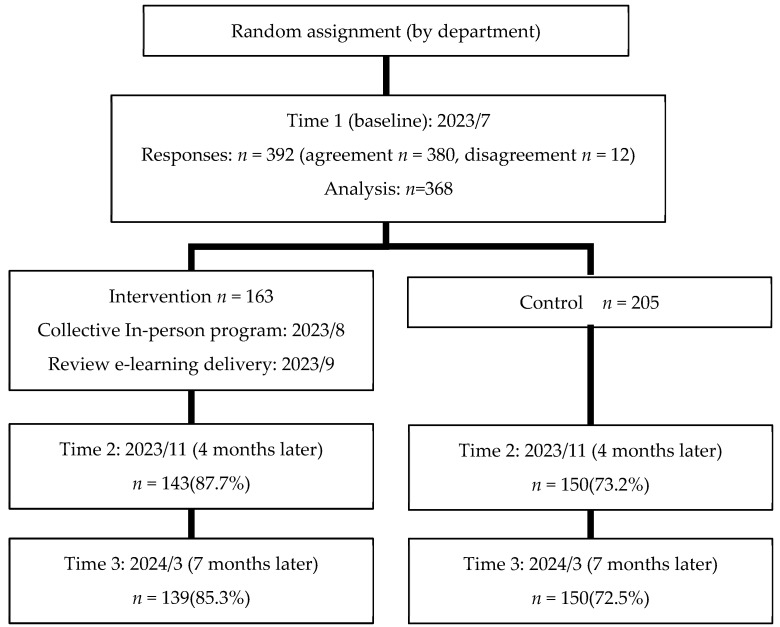
Flowchart of the research subjects.

Figure 1 shows a flowchart of the participants. The intervention program was delivered to the general employee group (*n* = 156) assigned to the intervention condition. Given that leadership traits and behavior are central to fostering psychological safety (A. C. Edmondson et al., 2004), a separate one-time training for all managers at Plant A was conducted in July 2023. This training focused on anger management and empathetic leadership, and its effectiveness was examined (Kobayashi et al., 2024). However, as it was conducted approximately a year before this study and included all managers, it was considered to have no differential effect on the intervention versus control group conditions in the present trial. Instead, the study deemed that equipping all managers with uniform knowledge and skills related to leadership traits and behaviors that form the foundation of psychological safety would enable it to control for differences among leaders. This approach was expected to elucidate the effects of the intervention targeting general employees.

### 2.2. Intervention Program

A face-to-face intervention program was implemented with general employees (*n* = 156) in the intervention group to equip them with coping strategies for interpersonal stress and enhance communication skills for expressing opinions to supervisors and colleagues (Table 2). To minimize burden and maximize feasibility, the program was designed as a one-time session. In this study, a 3 h face-to-face program was conducted by clinical and licensed psychologists, combining lecture, group work, individual tasks, and hands-on training. To reduce infection risk and minimize workflow disruption, sessions were held across six groups (20–30 participants each), all within the same week to ensure consistency in content and delivery.

The program consisted of four components. Program 1 introduced three interpersonal stress-coping strategies, followed by specific instruction and exercises in Programs 2 through 4. Program 2 focused on stress coping using cognitive-behavioral techniques. Participants worked through interpersonal stress examples to reframe unhelpful thought patterns into realistic, objective perspectives. They also explored their own thought habits and practiced mindfulness to regulate emotions such as anxiety and anger. Program 3 introduced self-care techniques, including breathing exercises and relaxation methods. Program 4 focused on assertion, a communication strategy for clearly expressing thoughts and feelings to reduce stressors. Participants explored four situational examples, including (1) making a difficult request, (2) giving clear guidance, (3) feeling under-recognized, and (4) asking a colleague to adjust work expectations, and discussed how to express themselves effectively using assertive techniques. Sample responses and key communication points were provided.

The program had two primary aims: (1) to enhance individual communication and stress-coping skills, which are key factors in psychological safety and (2) to serve as a shared learning space where colleagues gained mutual understanding of the importance of sincere and necessary communication through extensive group work and practical activities.

To reinforce learning and workplace application, an e-learning review video was sent to intervention participants about one month after the session. To prevent contamination, participants were asked not to share program content with those in the control group. Control group employees were informed that they would receive the same training after the study period concluded.

### 2.3. Questionnaire

#### 2.3.1. Individual Attributes

Participants provided demographic information, including self-identified gender (male, female), age, job position (general or managerial), department (1–14), years of service, and job type (production/technical, clerical, research/specialized, or other).

#### 2.3.2. Work Engagement

Work engagement was assessed using the Japanese short version of the Utrecht Work Engagement Scale (Shimazu et al., 2008), which consists of nine items across three subscales, including vigor (e.g., “I feel energized when I work”), dedication (e.g., “I feel proud of my work”), and absorption (e.g., “I am enthusiastic about my work”), with three items per subscale. A seven-point Likert scale (0 = “never” to 6 = “always/every day”) was used. Reliability and validity have been verified. Scores were averaged to compute an overall engagement score (range: 0–6), with higher scores indicating greater engagement.

#### 2.3.3. Experience of Workplace Bullying

The Workplace Power Harassment Perceptions and Experiences Scale (Nii et al., 2018) was used, developed in accordance with the Japanese definition of workplace bullying. Workplace bullying and power harassment share a common feature: the perpetrator’s words and actions lead to physical, mental, and social distress for the victim. While definitions of workplace bullying in western contexts require such words, actions, or situations to persist over a certain period, Japan lacks a specification of frequency or duration. Furthermore, the Japanese definition of power harassment emphasizes that it stems from various workplace superiorities (e.g., position, age, seniority, years of service, and experience/knowledge), involves actions that exceed the appropriate scope of work, and refers not only to acts that lead to mental or physical distress but also to those that weaken the quality of workplace environments. This scale was developed in accordance with Japan’s definition of power harassment and is a validated measure with confirmed reliability and validity. The scale comprises 18 items across three dimensions: (a) twelve items on bullying behaviors (e.g., “When angry, I lash out—punching, kicking, etc.,” or “Assigning clearly unnecessary tasks or withholding work out of dislike”), (b) four items on bullying situations (e.g., “Subordinates are intimidated by their superiors”), and (c) two items on bullying-related attitudes (e.g., “Superiors dismiss differing opinions or complaints”). Participants responded to two types of questions: (1) experience of workplace bullying over the past six months (1 = “never,” 2 = “once,” 3 = “repeatedly”) and (2) perception of exposure to bullying conditions (1 = “not applicable,” 2 = “applicable if repeated,” 3 = “applicable even once”). For analysis, subscale scores were averaged, resulting in bullying experience scores ranging from 0 to 3.

#### 2.3.4. Psychological Safety

The Japanese version of the Workplace Psychological Safety Scale (Sasaki et al., 2022) was used. This is a translated and validated version of the original Psychological Safety Scale (O’Donovan et al., 2020), with its target population expanded to include general workers. As such, all employees could serve as survey participants. The scale includes nine items related to team leaders or supervisors (e.g., “I can communicate my opinions about work-related problems to the team leader [or supervisor]”), seven items concerning colleagues and other team members (e.g., “I feel comfortable telling my colleagues about mistakes I have made in this team”), and three items referring to the team as a whole (e.g., “We can exchange information with each other about work problems in the team”). Subscale scores were calculated by dividing the total score of each section by the number of items in that section. The overall psychological safety score was calculated by dividing the combined total by 19, the total number of items.

#### 2.3.5. Coping Characteristics

The Brief Scale of Coping Profile for Workers (BSCP) was used. BSCP is an 18-item, six-subscale instrument developed to measure coping characteristics of workers under stress (Kageyama et al., 2004). Its reliability and validity have been confirmed. The subscales include active solutions (e.g., “I investigate causes and try to solve problems”), seeking help for solutions (e.g., “I consult with trusted people for solutions”), changing perspective (e.g., “I have hope that I can manage”), changing mood (e.g., “I distract myself with hobbies and entertainment”), and emotional expression involving others (e.g., “blaming the person who put me in that situation”), and avoidance and suppression (e.g., “letting go or postponing the problem”). These subscales reflect coping categories identified in previous research (Lazarus & Folkman, 1984). Higher scores indicate more frequent use of those coping behaviors.

### 2.4. Hypothesis

**H1.**  *General employees who participate in a group intervention program that equips them with coping strategies for interpersonal stress situations will exhibit (a) increased positive stress-coping strategies and (b) decreased victimization by power harassment and increased levels of work engagement and psychological safety.*

### 2.5. Analysis

IBM SPSS Statistics 28.0 was used for data analysis. To examine changes in work engagement, workplace bullying experiences (i.e., behaviors, attitudes, and conditions), psychological safety (i.e., supervisors, peers, and team), and BSCP subscales (i.e., active solutions, help-seeking, change in perspective, change in mood, emotional divergence, and avoidance/suppression), linear mixed model analyses were conducted. Analyses were stratified by job position (managerial vs. general) because the intervention was implemented only for general employees. This separation allowed us to isolate the program’s effects from any influence associated with managers who did not receive the intervention. Each outcome variable was treated as the dependent variable, with intervention condition (yes/no) and survey time point (Time 1, Time 2, Time 3) as fixed effects. The interaction between intervention status and time point was used to assess the intervention’s effect, specifically examining whether significant changes occurred from the baseline (Time 1) to post-intervention points (Time 2 and Time 3). In the linear mixed-effect model analysis, including subjects as random effects fundamentally accounts for individual-level variation. Furthermore, adding unnecessary covariates can destabilize parameter estimates and decrease a model’s interpretability; therefore, their inclusion was minimized in this study. However, it retained job position as a covariate given that the intervention program specifically targeted general employees. Thus, position was directly relevant to the interpretation of intervention effects, whereas other attributes (e.g., age, gender) were excluded. Additionally, a mediation analysis was conducted to explore which components of the intervention contributed to observed effects. In this analysis, intervention status (1 = present, 0 = absent) was the independent variable, psychological safety served as the mediator, and consultation for problem solving was the dependent variable. Indirect effects were evaluated using the bias-corrected bootstrap method with 2000 samples to calculate 95% confidence intervals (CI).

## 3. Results

### 3.1. Basic Analysis

Table 3 presents descriptive statistics for the intervention and control groups at each time point in the study.

### 3.2. Intervention Effects

Results of the linear mixed model analysis for each dependent variable are shown in Table 4.

#### 3.2.1. Work Engagement

No significant differences were found among managers. For general employees, the interaction between intervention presence and time of survey showed a trend toward significance (F(2, 689.42) = 2.95, *p* = 0.053). A simple main effect test revealed a significant effect of survey time in the control group (F(2, 381.73) = 6.07, *p* = 0.003). Bonferroni’s multiple comparison indicated significant decreases in work engagement at Time 2 and Time 3 compared to Time 1 (Time 2: *p* = 0.008, d = 0.29, 95% CI [−0.58, −0.07]; Time 3: *p* = 0.004, d = 0.28, 95% CI [−0.61, 0.10]). No significant main effects were observed in the intervention group.

#### 3.2.2. Workplace Bullying

Among managers, no significant effects were found. For general employees, a significant interaction was found for workplace bullying conditions (F(2, 666.55) = 9.46, *p* < 0.001). Simple main effect tests showed significant time effects in both intervention (F(2, 356.34) = 5.67, *p* = 0.004) and control groups (F(2, 304.97) = 4.44, *p* = 0.013). Bonferroni’s test revealed a significant decrease in workplace bullying conditions at Time 3 in the intervention group (*p* = 0.006, d = 0.28, 95% CI [−0.26, −0.04]), and a significant increase at Time 3 in the control group (*p* = 0.002, d = 0.23, 95% CI [0.05, 0.25]). No significant interaction was found for bullying behaviors or attitudes.

#### 3.2.3. Psychological Safety

For general employees, significant interactions were found for psychological safety toward supervisors (F(2, 653.55) = 7.25, *p* < 0.001) and teams (F(2, 637.88) = 5.27, *p* = 0.005). No significant results were observed for managers. Simple main effect testing showed a trend for the intervention group on supervisor-related psychological safety (F(2, 289.17) = 2.90, *p* = 0.056) and a significant effect for the control group (F(2, 341.91) = 5.15, *p* = 0.006). Psychological safety toward supervisors significantly increased at Time 3 in the intervention group (*p* = 0.033, d = 0.22, 95% CI [0.01, 0.43]) and decreased in the control group (*p* = 0.003, d = 0.22, 95% CI [−0.51, −0.09]). For team-level psychological safety, the intervention group showed a significant main effect (F(2, 291.01) = 3.23, *p* = 0.041), and the control group showed a near-significant trend (F(2, 340.36) = 2.56, *p* = 0.008). Psychological safety for the team significantly increased at Time 3 in the intervention group (*p* = 0.023, d = 0.20, 95% CI [0.03, 0.48]), while it significantly declined in the control group (*p* = 0.05, d = 0.14, 95% CI [−0.44, 0]). No significant interaction was found for psychological safety with coworkers.

#### 3.2.4. Coping Characteristics

No significant differences were found for managers. For general employees, significant interactions were found in consultation for problem solving (F(2, 653.65) = 4.64, *p* = 0.01) and mood regulation (F(2, 639.46) = 3.99, *p* = 0.019). Simple main effect tests showed a significant increase in consultation behaviors in the intervention group (F(2, 289.39) = 11.88, *p* < 0.001; *p* < 0.001, d = 0.30, 95% CI [0.13, 0.41]), with no significant change in the control group. For mood regulation, effects trended toward significance in both groups (intervention: F(2, 293.71) = 2.81, *p* = 0.062; control: F(2, 336.40) = 2.90, *p* = 0.057). Bonferroni’s test showed a marginal increase at Time 3 in the intervention group (*p* = 0.054, d = 0.18, 95% CI [−0.002, 0.29]) and at Time 2 (*p* = 0.066, d = 0.06, 95% CI [−0.01, 0.25]), though effect sizes were small. No significant interactions were found for other BSCP subscales.

In summary, the intervention program significantly improved consultation for problem solving among participating general employees. Psychological safety toward both supervisors and teams also increased, while experiences of workplace bullying, particularly intimidation by superiors, decreased. Although work engagement declined in the control group, it remained stable in the intervention group, suggesting the program helped maintain engagement, though it did not significantly improve it. Hypotheses 1-1 and 1-2 were partially supported.

### 3.3. Complementary Analysis

**Figure 2:**
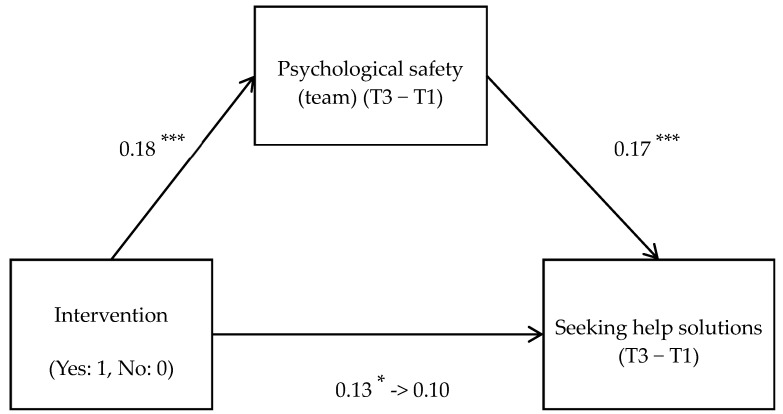
Effectiveness of intervention programs in mediating increased psychological safety and facilitating problem-solving counseling. Note. *** *p* < 0.001, * *p* < 0.05.

A mediation analysis was conducted to examine whether the intervention promoted consultation behaviors through improved psychological safety, or whether the intervention fostered consultation as a coping skill that then enhanced psychological safety. Results (Figure 2) indicated that the intervention’s effect on problem-solving consultation was fully mediated by psychological safety (b = 0.049, BootSE = 0.032, 95% CI [0.001, 0.109]). No significant mediation effect was found when the mediating variable was consultation and the dependent variable was psychological safety.

## 4. Discussion

This study developed an intervention program for general employees focused on building psychological safety to help prevent workplace bullying and enhance work engagement and evaluated its multifaceted effects through a cluster randomized controlled trial. The findings showed that the program increased consultation behavior for problem solving as a stress-coping strategy, enhanced psychological safety toward superiors and teams, and reduced experiences of workplace bullying, characterized by excessive fear of superiors and withdrawal. Consistent with these outcomes, the analyses largely supported Hypotheses 1a and 1b. Notably, the intervention did not improve stress coping first and then led to psychological safety; rather, psychological safety increased first, which then led to more consultative behaviors. General employees who participated in the program became more comfortable expressing opinions without excessive fear or hesitation toward supervisors and team members. Their tendency to consult others and take proactive steps in stressful situations also increased. These outcomes likely reflect not only improved individual communication skills but also broader organizational effects, as the group-based format allowed participants to exchange ideas and foster mutual understanding in real time. Employees who underwent the program developed a shared understanding of the importance of and strategies for expressing the need for consultations, requests, and reports candidly, and it sincerely demonstrates the emergence of a team-level belief: honest consultations will not threaten interpersonal relationships. In turn, doing so increased individual coping, specifically by consulting others to resolve issues. This aspect aligns with the conceptual definition of psychological safety as a belief shared within groups (e.g., teams or organizations) instead of solely a personal cognitive aspect (A. C. Edmondson & Lei, 2014).

It is believed the program contributed to psychological safety and reduced workplace bullying by helping participants recognize the importance of open and sincere communication regarding consultations, requests, and reporting, along with strategies for doing so. While the intervention included several components, the assertion module may have played a key role, given its direct relevance to psychological safety, bullying prevention, and problem-solving consultation. However, this study was unable to definitively establish this notion. Future studies should evaluate the specific contributions of individual modules to determine which elements are most effective. Although work engagement significantly declined in the control group, it remained stable in the intervention group, suggesting that the program may have buffered against decline, even if it did not enhance engagement. External organizational factors could have contributed to low initial work engagement in the intervention group. Since the COVID-19 pandemic, employee engagement, particularly in small- to medium-sized enterprises with fewer than 900 employees, has been declining (Persol Research and Consulting Co., Ltd., 2025), and the company under study may have also been affected. This aspect implies that the program may function as a protective buffer against adverse external conditions (not limited to COVID-19–related disruptions) that suppress work engagement among employees. Thus, maintaining engagement may constitute a meaningful effect of the program under the current conditions.

### 4.1. Theoretical Implications

The findings extend and refine three complementary perspectives in several ways: psychological safety theory, the JDR model, and stress and coping theory. First, with respect to psychological safety theory, the results of mediation analysis pointed to a pattern consistent with the function of psychological safety as a proximal team-climate mechanism that preceded and encouraged consultation to achieve problem resolution. Consultation-seeking may increase when members previously perceive safety, which is in contrast with improving consultation skills and subsequently enhancing safety. This pattern aligns with the theorized role of psychological safety as an immediate facilitator of self-expression, help-seeking, and learning (A. Edmondson, 1999; A. C. Edmondson & Lei, 2014). Notably, the present brief, group-delivered program targeted general employees instead of managers alone but, nevertheless, enhanced perceptions of psychological safety toward supervisors and teams across several months. This finding indicates a practical extension of psychological safety theory: equipping members with interpersonal skills and norms that support interpersonal risk-taking can foster shared perceptions of safety.

Second, for the JDR model, the observation that self-reported experiences with bullying decreased while psychological safety increased, and engagement was preserved in the intervention group but declined in the control group, is consistent with the function of psychological safety as a team-level job resource that buffers engagement against adverse conditions (i.e., bullying as a hindrance demand). This aspect provides field-experimental support for the propositions of the JDR regarding resource–demand dynamics and identifies psychological safety as a resource that can be strengthened through brief, skill-based interventions at the unit level (Demerouti et al., 2001; Bakker & Demerouti, 2007). The findings also converge with evidence that broad psychosocial safety climates are associated with decreased bullying risk and improved well-being, which positions the proposed mechanism at the proximal (team) level within a multilevel climate perspective (Law et al., 2011).

Lastly, for stress and coping theory, the program explicitly provided training on cognitive reframing, self-regulation, and assertive communication. However, the mediation pattern implies that coping behaviors were more fully activated when social appraisal signaled safety—specifically, when members believed that they could express themselves without excessive interpersonal risk. This finding refines coping perspectives by highlighting that appraisals of controllability and available responses depend not only on individual skills but also on climate indicators of interpersonal risk (Lazarus & Folkman, 1984). Therefore, climate and coping appear to be mutually enabling, whereas psychological safety emerges as a proximal gatekeeper of the extent of coping.

Taken together, the results support an integrated pathway that links micro-level skills to meso-level climate and outcomes: unit level, skills-based training → shared norms and interpersonal efficacy → greater psychological safety (toward supervisors and teams) → increased consultation/help-seeking → fewer perceived bullying conditions → preserved engagement under ambient stress. This framework elucidates why even a brief, one-session program may produce measurable changes in shared perceptions and behaviors while indicating that direct improvements in engagement may require longer intervals, which is consistent with the “resource–gain” processes in the JDR model. Thus, future trials should test these delayed effects and examine cross-level interactions (e.g., manager behaviors × member skills) to further refine the multilevel integration of psychological safety, JDR, and coping perspectives.

### 4.2. Practical Implications

These findings pose important implications for practice and policy. At the organizational level, this study demonstrates that psychological safety can be actively cultivated using targeted, skill-based interventions and is closely linked to individual resilience. Therefore, such programs should be integrated into routine training to prevent workplace bullying and foster a culture of open communication and collective problem-solving. Given the stability of work engagement in the intervention group despite broad downward trends, these programs may serve as a valuable buffer in scenarios that induce organizational stress or economic uncertainty. Scaling this type of evidence-based intervention across diverse sectors and company sizes could contribute to extensive improvements in well-being and productivity among employees.

In terms of practicality, HR and training departments could implement the program through a brief group session (approximately 3 h with 20–30 employees) reinforced by a brief e-learning follow-up. Embedding the program in intact work units could help establish shared norms, thereby extending psychological safety from individual skills to team climate. In Japan, many practical initiatives to promote psychological safety have primarily focused on manager training; however, the current study highlights the value of involving general employees and the broad workforce in recognizing the importance of open and sincere communication and developing concrete skills to practice this type of communication.

### 4.3. Limitations

This study has several limitations. Firstly, reliance on self-reported data may have introduced bias, and the brief follow-up period limited conclusions regarding long-term effects on engagement. Future research should employ multiple data collection methods and extend the follow-up period to evaluate sustained outcomes. Secondly, the sample was drawn from a single company at one site, which limits generalizability despite allowing control over contextual factors. Future studies should therefore include multiple companies and sites to enhance external validity. Thirdly, observations of engagement effects were not entirely clear, although prior research in Japan has demonstrated delayed improvements after six months (Imamura et al., 2017). Longer-term studies are needed to verify whether such delayed effects emerge. Finally, although the cRCT design minimized contamination and reflected the intervention’s group-level nature, it also reduced statistical power due to intra-cluster correlation and potential imbalance. These methodological constraints should be addressed in future research through larger sample sizes and stratified randomization.

## 5. Conclusions

This cRCT of a brief, group-based training for general employees demonstrated improved psychological safety, reduced bullying conditions, increased consultation-seeking for problem solving, and sustained engagement during a period of decline, with mediation analysis suggesting that enhanced psychological safety preceded and enabled consultation. Theoretically, the findings position psychological safety as a proximal lever for sustained bullying prevention and engagement, while practically, they provide a feasible implementation model—one three-hour session plus e-learning with intact teams—that organizations can incorporate into routine organizational development. Although limited by self-report measures, a single site, and brief follow-up, this study retains theoretical and practical significance by offering guidance for scalable, culture-shaping interventions.

## Figures and Tables

**Table 1:** Participant attributes (age and length of service are shown as mean ± 1 SD).

	Intervention (*n* = 163)		Control (*n* = 205)	
	*n*	%	*n*	%
Gender				
Male	81	50	93	45
Female	82	50	112	55
Age	38.76 ± 11.84		36.87 ± 11.70	
Position				
General	156	96	194	95
Management	7	4	11	5
Years of service	11.56 ± 10.24		10.44 ± 9.12	
Job Type				
Production and engineering	122	75	147	72
Administrative	21	13	27	13
Research/specialized	1	1	7	3
Other	19	12	24	12
Department				
1			31	15
2	44	27		
3			45	22
4	12	7		
5			50	24
6	61	37		
7	25	15		
8			5	2
9			39	19
10	21	13		
11			19	9
12			10	5
13			4	2
14			2	1
Total	163	100	205	100

**Table 2:** Overview of the intervention program (participants: general staff in the intervention group, *n* = 156).

	Program (Minutes)	Summary
1	Stress Management (30)	Provides a conceptual understanding of workplace stress and introduces basic strategies for stress coping. As an introduction to the full program, it outlines three key approaches: increasing stress tolerance (Program 2), relieving accumulated stress (Program 3), and reducing stressors (Program 4). (Lecture and individual work)
2	Cognitive-behavioral therapy and mindfulness for stress resilience (60)	Introduces both cognitive and physical coping methods based on cognitive-behavioral therapy. Aims to foster objective perspectives on interpersonal stress, emotional awareness, and self-regulation. Participants explore personal thought patterns and emotional tendencies to deepen self-understanding. The session also promotes mindfulness practice to enhance stress tolerance, emotional control, and empathy. (Lecture, individual/group work, and practical training)
3	Relaxation and coping behaviors for stress reduction (20)	Introduces self-care techniques such as breathing exercises and typical relaxation methods, along with behaviors aimed at relieving stress. (Lecture and practical training)
4	Assertion, the art of communication that values both the self and others (65)	Develops assertive communication skills, expressing opinions, requests, and emotions sincerely and respectfully. Focuses on skills necessary for effective workplace interaction, including reporting, consultation, requests, and instruction. (Lecture and group work)

**Table 3:** Basic statistics at each time point for the intervention and control groups.

	Intervention						Control					
	Time 1 (*n* = 163)		Time 2 (*n* = 154)		Time 3 (*n* = 152)		Time 1 (*n* = 205)		Time 2 (*n* = 190)		Time 3 (*n* = 194)	
	M	SD	M	SD	M	SD	M	SD	M	SD	M	SD
Work engagement	2.43	1.28	2.51	1.28	2.48	1.29	2.56	1.16	2.28	1.26	2.29	1.31
Workplace bullying												
Workplace bullying (behaviors)	1.14	0.29	1.10	0.21	1.11	0.26	1.10	0.25	1.06	0.15	1.08	0.21
Workplace bullying (attitude)	1.30	0.59	1.20	0.48	1.22	0.50	1.15	0.44	1.14	0.40	1.15	0.40
Workplace bullying (situations)	1.34	0.58	1.27	0.54	1.20	0.46	1.28	0.54	1.34	0.58	1.41	0.64
Workplace bullying (total)	1.26	0.42	1.19	0.33	1.18	0.31	1.18	0.35	1.18	0.32	1.21	0.34
Psychological safety												
Psychological safety (supervisor)	4.69	1.33	4.82	1.38	4.97	1.37	4.88	1.29	4.84	1.38	4.61	1.41
Psychological safety (colleagues)	5.13	1.28	5.17	1.34	5.25	1.34	5.15	1.24	5.20	1.21	5.08	1.26
Psychological safety (team)	4.66	1.43	4.80	1.42	4.96	1.46	5.00	1.39	4.90	1.42	4.81	1.41
Psychological safety (total)	4.85	1.18	4.94	1.20	5.07	1.25	5.00	1.14	4.98	1.17	4.82	1.18
Coping												
Active solutions	2.97	0.73	2.91	0.80	3.05	0.77	2.86	0.74	2.89	0.69	2.94	0.69
Seeking help for solutions	2.74	0.85	2.75	0.88	3.00	0.85	2.81	0.84	2.84	0.72	2.86	0.80
Changing mood	2.59	0.87	2.65	0.84	2.74	0.96	2.75	0.88	2.81	0.84	2.73	0.89
Emotional expression involving others	1.66	0.54	1.78	0.54	1.80	0.56	1.66	0.47	1.71	0.51	1.69	0.48
Avoidance and suppression	1.97	0.75	2.03	0.72	2.05	0.77	1.95	0.72	2.09	0.76	1.96	0.66
Changing perspectives	2.21	0.74	2.28	0.72	2.24	0.69	2.30	0.68	2.20	0.71	2.27	0.70

Note: SD, standard deviation; M, Mean.

**Table 4:** Results of the linear mixed model analysis.

		T1	T2	T3	Simple Main Effect		Bonferroni			
	Intervention	*Mean*			*F*				*d*	95% CI
Work Engagement	Presence	2.45	2.47	2.45	0.03	n.s	-		-	-
	Absence	2.56	2.23	2.20	6.07	**	T1 > T2 T1 > T3	** **	0.29 0.28	[−0.58, −0.07] [−0.61, 0.10]
workplace bullying (situations)	Presence	1.35	1.28	1.20	5.67	**	T1 > T3	**	0.28	[−0.26, −0.04]
	Absence	1.28	1.34	1.43	4.44	*	T1 < T3	**	0.23	[0.05, 0.25]
Psychological safety (supervisor)	Presence	4.69	4.79	4.91	2.90	^†^	T1 < T3	*	0.22	[0.01, 0.43]
	Absence	4.86	4.75	4.56	5.15	**	T1 > T3	**	0.22	[−0.51, −0.09]
Psychological safety (team)	Presence	4.68	4.81	4.94	3.23	*	T1 < T3	*	0.20	[0.03, 0.48]
	Absence	4.97	4.85	4.75	2.56	^†^	-		0.14	[−0.44, 0]
seeking help solutions	Presence	2.74	2.75	3.01	11.88	***	T1 < T3	***	0.30	[0.13, 0.41]
	Absence	2.81	2.82	2.85	0.22	n.s	-		-	-
changing mood	Presence	2.63	2.65	2.77	2.81	^†^	-		0.18	[−0.002, 0.29]
	Absence	2.74	2.86	2.74	2.90	^†^	-		0.06	[−0.01, 0.25]

Note. *** *p* < 0.001, ** *p* < 0.01, * *p* < 0.05, ^†^ *p* < 0.10.

## Data Availability

The data supporting the findings of this study are not publicly available due to privacy concerns and confidentiality agreements. Access is restricted to protect the participants’ personal information in accordance with ethical guidelines.
